# Application of dry olive residue-based biochar in combination with arbuscular mycorrhizal fungi enhances the microbial status of metal contaminated soils

**DOI:** 10.1038/s41598-022-17075-5

**Published:** 2022-07-25

**Authors:** José A. Siles, Inmaculada García-Romera, Tomas Cajthaml, Jorge Belloc, Gloria Silva-Castro, Jirina Szaková, Pavel Tlustos, Mercedes Garcia-Sanchez

**Affiliations:** 1 Department of Plant & Microbial Biology, University of California at Berkeley, Berkeley, CA USA; 2 Department of Soil Microbiology and Symbiotic Systems, Estación Experimental del Zaidín, Consejo Superior de Investigaciones Científica (EEZ-CSIC), Granada, Spain; 3 Institute of Microbiology of the Academy of Sciences, Prague, Czech Republic; 4 Faculty of Science, Institute for Environmental Studies, Charles University, Prague, Czech Republic; 5 Department of Agro-Environmental Chemistry and Plant Nutrition, Faculty of Agrobiology, Food and Natural Resources, Prague, Czech Republic; 6 Eco&Sols, CIRAD, INRAE, IRD, Institut Agro Montpellier, Université Montpellier, Montpellier, France

**Keywords:** Microbiology, Microbial communities, Environmental microbiology, Environmental sciences, Environmental impact

## Abstract

Biochar made-up of dry olive residue (DOR), a biomass resulting from the olive oil extraction industry, has been proposed to be used as a reclamation agent for the recovery of metal contaminated soils. The aim of the present study was to investigate whether the soil application of DOR-based biochar alone or in combination with arbuscular mycorrhizal fungi (AMF) leads to an enhancement in the functionality and abundance of microbial communities inhabiting metal contaminated soils. To study that, a greenhouse microcosm experiment was carried out, where the effect of the factors (i) soil application of DOR-based biochar, (ii) biochar pyrolysis temperature (considering the variants 350 and 500 °C), (iii) soil application dose of biochar (2 and 5%), (iv) soil contamination level (slightly, moderately and highly polluted), (v) soil treatment time (30, 60 and 90 days) and (vi) soil inoculation with *Funneliformis mosseae* (AM fungus) on β-glucosidase and dehydrogenase activities, FA (fatty acid)-based abundance of soil microbial communities, soil glomalin content and AMF root colonization rates of the wheat plants growing in each microcosm were evaluated. Biochar soil amendment did not stimulate enzyme activities but increased microbial abundances. Dehydrogenase activity and microbial abundances were found to be higher in less contaminated soils and at shorter treatment times. Biochar pyrolysis temperature and application dose differently affected enzyme activities, but while the first factor did not have a significant effect on glucosidase and dehydrogenase, a higher biochar dose resulted in boosted microbial abundances. Soil inoculation with *F. mosseae* favored the proliferation of soil AMF community and increased soil glomalin content as well as rates of AMF root colonization. This factor also interacted with many of the others evaluated to significantly affect soil enzyme activities, microbial abundances and AMF community. Our results indicate that the application of DOR-based biochar along with AMF fungi is an appropriate approach to improve the status of microbial communities in soils with a moderate metal contamination at short-term.

## Introduction

Contamination by metals has generally chronic consequences for soils since most of these pollutants are not subjected to microbial or chemical degradation and remain in these ecosystems for long-lasting periods, compromising the quality services provided by soils and negatively impacting soil life and its functions^1^. From a microbial perspective, metals have shown to have a detrimental impact on soil microbial functionality^2,3^, abundance^4,5^ and taxonomic diversity^6^. The extent of this impact is dependent on the type and level of metal contamination and the type of soil considered^4,5^. Many soils chronically contaminated with metals are characterized by harboring microbial communities with a poor microbial activity and taxonomically restricted to metal-resistant groups, which limits their functional capabilities^7,8^. Soil microorganisms can be used as indicators of ecosystem quality because of their capability to quickly respond to environmental shifts. Likewise, they play a pivotal role in the functioning of the entire ecosystem as they are involved in soil organic matter (SOM) decomposition, nutrient cycling, maintaining soil structure and stablishing symbiotic relationships with plants^9,10^. Therefore, effective reclamation treatments of metal polluted soils should not only include strategies to reduce the bioavailability of metals, but also to enhance the status of the autochthonous microbial communities.

Biochar has attracted great attention to be used as a reclamation agent for metal contaminated soils in the last few years^11^. This carbon-rich material is obtained through pyrolysis and is characterized by having an alkaline pH, a porous structure, a large surface area, a high cation exchange capacity (CEC) and abundant surface functional groups^12,13^. Biochar can directly (through complexation, cation exchange, electrostatic interactions, reduction and precipitation processes) or indirectly (through affecting soil pH, CEC and SOM contents) reduce the mobility and bioavailability of metals and their transfer to other ecosystem as well as their uptake by plants^14,15^. Biochars based on municipal and animal wastes, wood, crop residues as well as biosolids have shown to reduce the bioavailability of metals in contaminated soils, as reviewed by Hean et al.^16^ and Yuan et al.^17^. However, in most of the cases, it is not well known whether the metal immobilization mediated by biochar is concomitant with an enhancement of the soil microbial status. In this way, integrative studies such as that of Xu et al.^18^, showing that biochar made of macadamia nutshell reduced soil Cd and Pb bioavailability and increased microbial respiration, abundance and carbon use efficiency, should be performed more commonly. Likewise, it is important to assess how factors that affect the reclamation effectiveness of biochar such as the inherent properties of the starting material, pyrolysis conditions (temperature and time), amendment doses, mixing depth, soil properties and climate conditions^13,14^ impact soil microbial communities during reclamation processes.

Arbuscular mycorrhizal fungi (AMF) establish symbiotic relationships with plants and expand the interface between plants and soil environment^19^. AMF ameliorate plant metal stress and enhance plant abilities in terms of nutrient and water uptake as well as pathogen resistance^20,21^ in different degrees depending on the AMF species considered^20^. This microbial group also plays an important role in reducing soil metal bioavailability by binding metals to the fungal structures and by complexing the pollutants through glomalin, an extracellular glycoprotein produced by all the AMF^22^. Metals, especially at high levels, can negatively affect the germination of AMF spores, the growth of extraradical mycelium and the mycorrhizal colonization^19,23^. The initial successful establishment of AMF symbiosis may thus be challenging in contaminated soils. The application of organic amendments such as biochar has shown to enhance AMF performance by increasing the availability of nutrients, changing soil physicochemical properties and improving spore germination^24^. Additionally, a number of studies have reported that AMF inoculation and biochar application have synergistic effects on promoting soil fertility and plant yield in metal contaminated soils^25^.

In the Mediterranean region, the olive oil industry produces huge amounts of an organic-matter-rich waste called dry olive residue (DOR), which has been proposed to be used as reclamation agent for metal polluted soils^26^. As it was demonstrated by Hovorka et al.^27^, DOR, especially after being transformed by saprotrophic fungi, has capabilities to absorb Cd, Zn and Pb. However, desorption experiments showed that metals were weakly and unstably immobilized. In this context, it has been hypothesized that the conversion of DOR into biochar may improve the capabilities of DOR to keep metals bound to a higher extent. Additionally, soil application of fungal transformed-DOR in combination with AMF is a viable practice to restore microbial functionality and biomass in soils chronically contaminated with metals^28^. Integrative studies assessing the potential of DOR-based biochar along with AMF application to reduce the bioavailability of metals and to enhance the activity and abundance of microbial communities in chronically metal polluted soils are missing. It has been recently found that DOR-based biochar decreased available contents of Cd and produced an ambiguous effect on the mobility of As, Pb and Zn in three soils presenting a pollution gradient collected from the same location^15^. We aimed in the present work at investigating whether the addition of DOR-based biochar alone or in combination with the AMF *Funneliformis mosseae* leads to an enhancement in the functionality and abundance of microbial communities inhabiting metal contaminated soils in concomitance with an alleviation of the metal mobility.

## Results

### Evaluation of the factors DOR-based biochar application, soil contamination level, soil treatment time, *F. mosseae* inoculation and their interactions throughout the experiment

**Figure 1:**
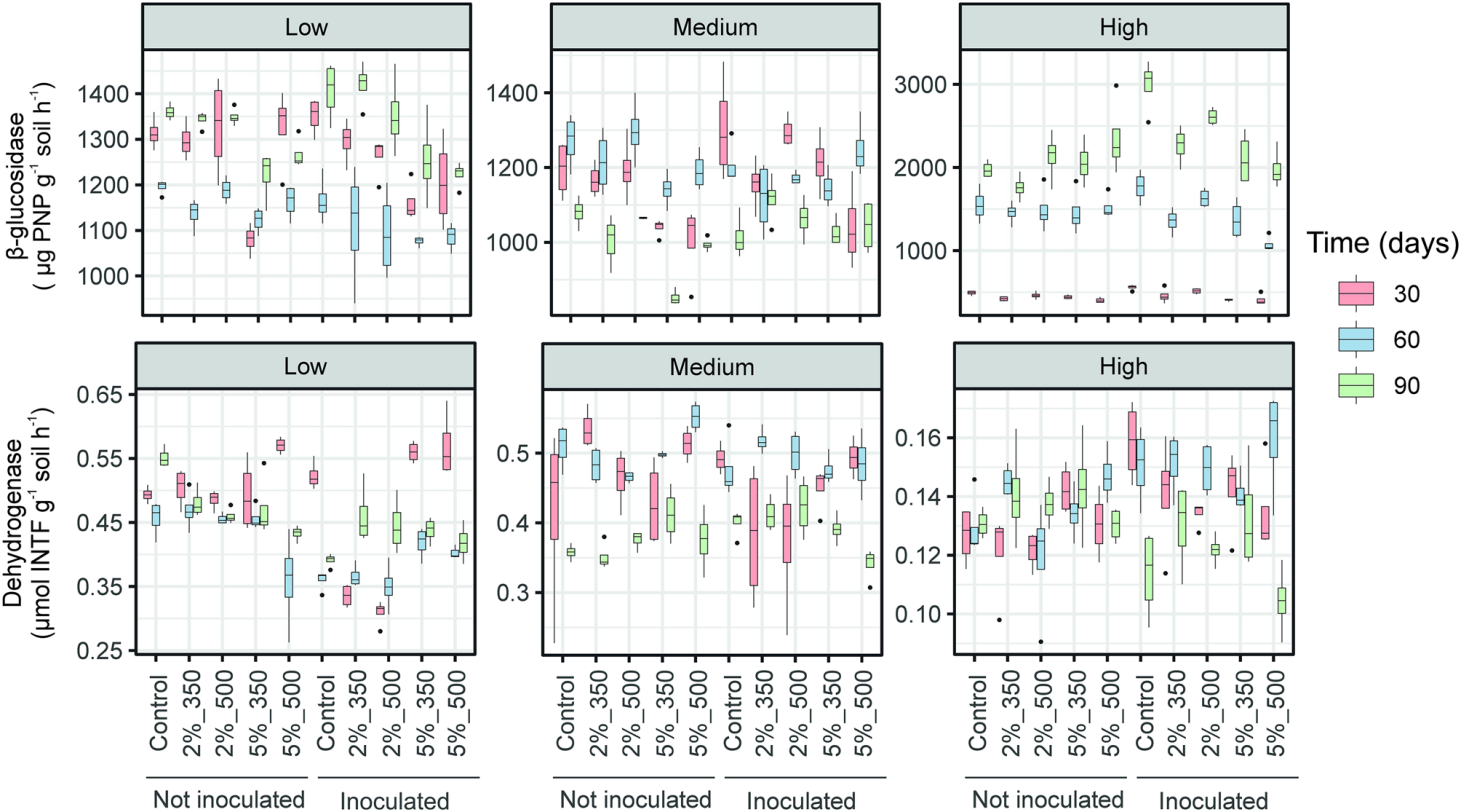
Box plots showing levels of β-glucosidase and dehydrogenase activities in soils contaminated at low, medium and high levels and amended at the doses of 2 and 5% with DOR-based biochar produced at 350 and 500 °C after 30, 60 and 90 days of experiment. Controls refers to unamended soils. Soils were inoculated with the AMF *F. mosseae*. The boxes represent the interquartile range (IQR) between the first and third quartiles (25th and 75th percentiles, respectively) and the vertical line inside the box defines the median. Whiskers represent the lowest and highest values within 1.5 times the IQR from the first and third quartiles, respectively. Dots represent outliers.

**Figure 2:**
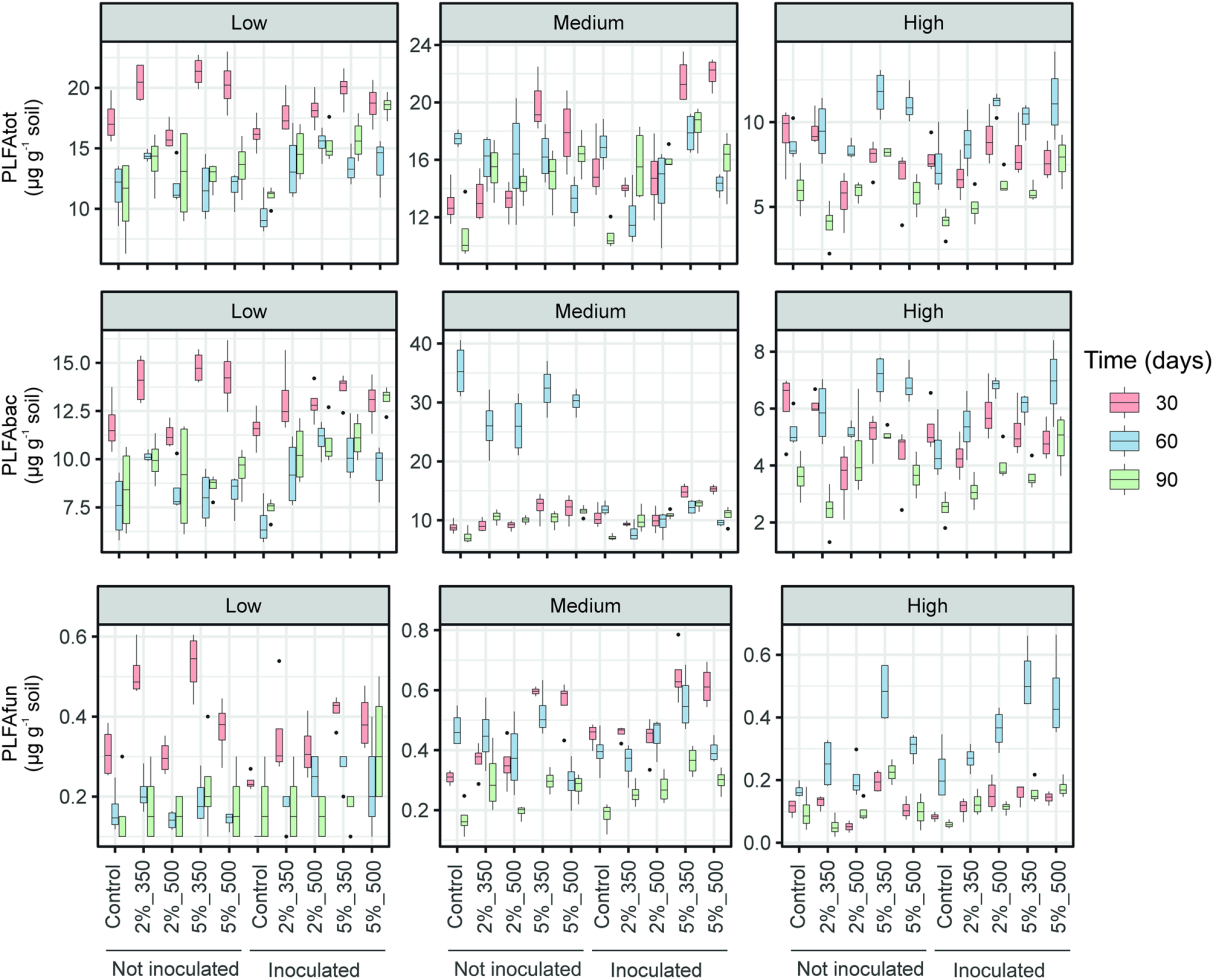
Box plots showing PLFA-based total, bacterial and fungal abundance in soils contaminated at low, medium and high levels and amended at the doses of 2 and 5% with DOR-based biochar produced at 350 and 500 °C after 30, 60 and 90 days of experiment. Controls refers to unamended soils. Soils were inoculated with the AMF *F. mosseae*. The boxes represent the interquartile range (IQR) between the first and third quartiles (25th and 75th percentiles, respectively) and the vertical line inside the box defines the median. Whiskers represent the lowest and highest values within 1.5 times the IQR from the first and third quartiles, respectively. Dots represent outliers.

**Figure 3:**
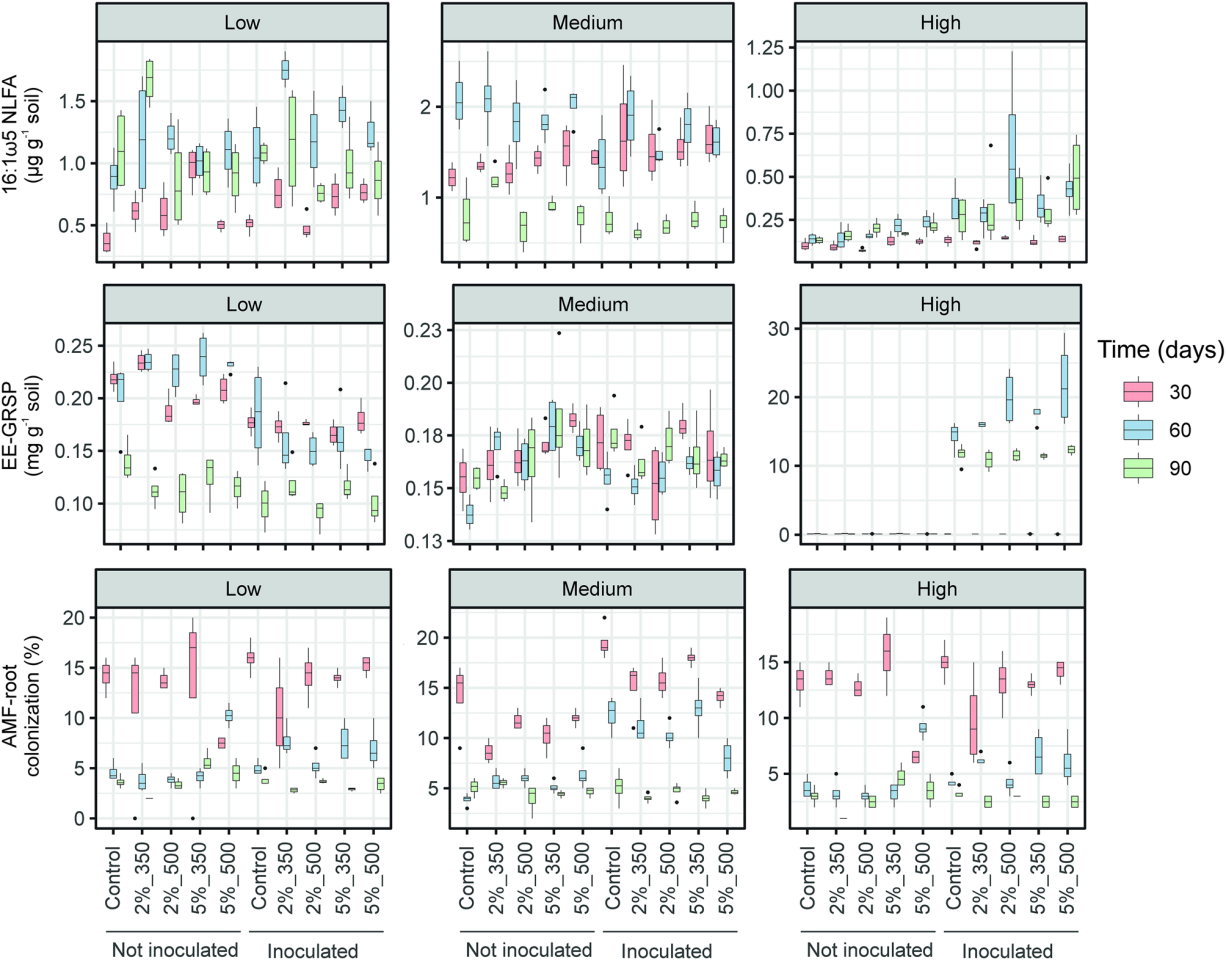
Box plots showing levels of NLFA-based abundance of AMF, easily extractable glomalin-related soil content (EE-GRSP) and AMF-root colonization rate of wheat plants grown in soils contaminated at low, medium and high levels and amended at the doses of 2 and 5% with DOR-based biochar produced at 350 and 500 °C after 30, 60 and 90 days of experiment. Control refers to unamended soils. Soils were inoculated with the AMF *F. mosseae*. The boxes represent the interquartile range (IQR) between the first and third quartiles (25th and 75th percentiles, respectively) and the vertical line inside the box defines the median. Whiskers represent the lowest and highest values within 1.5 times the IQR from the first and third quartiles, respectively. Dots represent outliers.

In a first analysis, we evaluated the effect of the factors soil application of DOR-based biochar (B), soil contamination level (S), soil treatment-time (T), *F. mosseae* (M) inoculation and their interactions on the status of soil microbial communities (soil enzyme activities, FA-based microbial abundance), EE-GRSPcontent and AMF root colonization percentage (Table 1, Figs. 1, 2, 3). The soil enzyme activities assayed, namely β-glucosidase and dehydrogenase, were differently influenced by the factors considered and their interactions. Specifically, β-glucosidase activity was reduced after soil amendment with DOR-based biochar, while dehydrogenase was not significantly affected. Soil contamination level was a significant factor for both activities, with the least contaminated soil showing significantly higher β-glucosidase and dehydrogenase activities throughout the experiment. Interestingly, the interaction between biochar application and soil contamination level was significant only for the β-glucosidase activity (B × S, p < 0.001). Longer treatment times led to a significant increase and decrease in β-glucosidase and dehydrogenase activities, respectively. The inoculation of *F. mosseae* boosted the soil β-glucosidase activity, which was also affected by the interaction between *F. mosseae* inoculation and biochar application (B × M, p < 0.05). On the other hand, although the AM fungus presence or absence did not significantly modify the dehydrogenase activity, the interaction of this factor with the soil treatment time and the factor biochar application or soil contamination level was significant (B × T × M, p < 0.001; S × T × M, p < 0.001, Table 1, Fig. 1).

**Table 1:** Results of MANOVA and post-hoc analyses on the effect of the factors application of DOR-based biochar, soil contamination level (“low”, “medium” and “high”), soil treatment time (30, 60 and 90 days), inoculation of *F. mosseae* and their interactions on soil enzyme activities (β-glucosidase and dehydrogenase), PLFA- and NLFA-based microbial abundance, easily extractable glomalin-related soil protein content (EE-GRSP) and AMF-root colonization rate.

Factors	β-glucosidase^a^	Dehydrogenase^b^	PLFA_tot_^c^	PLFA_bac_^c^	PLFA_Gram+_^c^	PLFA_Gram-_^c^	PLFA_act_^c^	PLFA_fun_^c^	NLFA_AMF_^c^	EE-GRSP^d^	AMF-root colonization^e^
**Biochar application (B)**											
F^significance^ (df)^f^	47.15*** (1)	0.19 (1)	36.04*** (1)	33.1*** (1)	11.44*** (1)	75.5*** (1)	8.74*** (1)	47.00*** (1)	13.48*** (1)	2.70 (1)	1.83 (1)
Post-hoc test											
No application	1283.79 b	0.39 a	10.54 a	7.02 a	2.67 a	2.87 a	1.07 a	0.17 a	0.63 a	0.16 a	5.00 a
Application	1191.40 a	0.39 a	13.80 b	9.44 b	3.26 b	4.10 b	1.25 b	0.28 b	0.77 b	0.16 a	6.00 a
**Soil contamination level (S)**											
F^significance^ (df)^f^	18.65*** (2)	2251.15*** (2)	314.44*** (2)	495.05*** (2)	258.73*** (2)	340.47*** (2)	555.19*** (2)	125.60*** (2)	872.95*** (2)	2553.75*** (2)	29.00*** (2)
Post-hoc test											
Low	1459.21 b	0.45 b	14.95 b	10.53 b	4.11 b	4.33 b	1.54 c	0.20 b	0.87 b	0.17 a	7.00 b
Medium	1127.80 a	0.46 b	15.25 b	10.95 c	3.85 b	4.96 c	1.31 b	0.38 c	1.36 c	0.16 a	9.00 c
High	1239.45 a	0.14 a	7.93 a	4.96 a	1.73 a	2.26 a	0.58 a	0.15 a	0.16 a	0.19 b	5.00 a
**Soil treatment time (T)**											
F^significance^ (df)^f^	485.17*** (2)	14.68*** (2)	52.40*** (2)	81.44*** (2)	14.00*** (2)	98.24*** (2)	19.41*** (2)	55.25*** (2)	64.06*** (2)	821.10*** (2)	362.20*** (2)
Post-hoc test											
30 days	1119.52 a	0.45 b	14.96 c	10.13 b	3.29 b	5.20 c	1.30 c	0.32 b	0.58 a	0.17 a	14.00 c
60 days	1196.07 b	0.42 b	12.62 b	8.55 b	2.98 b	3.80 b	1.23 b	0.30 b	1.16 c	0.19 c	5.75 b
90 days	1341.95 c	0.38 a	12.54 a	8.58 a	2.50 a	3.34 a	1.17 a	0.20 a	0.70 b	0.15 b	3.55 a
***F. mosseae* inoculation (M)**											
F^significance^ (df)^f^	8.81*** (1)	2.62 (1)	0.74 (1)	23.97*** (1)	3.85 (1)	1.66 (1)	5.57* (1)	1.07 (1)	24.56*** (1)	3122.76*** (1)	24.29*** (1)
Post-hoc test											
No inoculation	1204.16 a	0.43 a	12.79 a	8.90 b	3.07 a	3.78 a	1.19 b	0.23 a	0.78 a	0.16 a	5.00 a
Inoculation	1270.64 b	0.33 a	13.09 a	8.89 a	3.16 a	4.13 a	1.16 a	0.29 a	0.86 b	3.41 b	8.57 b
Significant interactions	B × S***, B × M*, S × T***, S × M***, T × M***, B × S × M***, S × T × M*	S × T***, S × M***, T × M*, B × T × M***, S × T × M***	B × T***, B × M***, S × T***, B × S × T***	B × T***, B × M***, S × T***, S × M***, M × T***, B × S × T***, S × T × M***	B × T***, S × T***, S × M***, B × S × T***, S × T × M***	B × T***, B × M***, S × T***, B × S × T***	B × S***, B × T***, B × M***, S × T***, S × M***, B × S × T*	B × M***, S × T***, B × S × T*	S × T***, S × M***, B × S × T*, S × T × M***	B × S*, S × T***, S × M***, T × M***, S × T × M***, B × S × T × M***	B × T***, S × T***, S × M***, M × T***, S × T × M*

Median values for each variable at each factor level are also shown.

^a^For post-hoc tests, the values are expressed as µPNP (g soil dm)^−1^ (1 h)^−1^.

^b^For post-hoc tests, the values are expressed as μmol INTF (g soil dm)^−1^ (1 h)^−1^.

^c^For post-hoc tests, the values are expressed as µg PLFA or NLFA (g soil dm)^−1^.

^d^For post-hoc tests, the values are expressed as mg (g soil dm)^−1^.

^e^For post-hoc tests, the values are expressed as %.

^f^F-value^significance^ (degrees of freedom).

For MANOVA analyses, F-values in bold denote statistical significance (p ≤ 0.05), significance levels are shown at *p < 0.05, **p < 0.01 and ***p < 0.001; for post-hoc Tukey’s HSD tests, median values of followed by different letters are significantly different (p < 0.05).

All the PLFA-based microbial fractions were significantly boosted by the soil amendment with DOR-based biochar (Table 1). Soil contamination level and treatment time were also significant factors for all the groups of PLFA markers, with soils “low” and “medium” showing significantly higher microbial abundances than the soil “high” and with the highest biomass contents being obtained after 30 and 60 days of the experiment. The microbial communities inhabiting the three soils responded to biochar addition in terms of abundance in a time-dependent manner as demonstrated by the significance of the interaction B × S × T for all the microbial fractions (Table 1, Fig. 2). A decline in the population of bacteria and actinobacteria was found in response to *F. mosseae* inoculation and this factor resulted not to be significant for the rest of PLFA-based microbial groups. Yet, the interaction between soil biochar addition and AMF application (B × M) had a significant effect on PLFA_tot_, PLFA_bac_, PLFA_Gram-_, PLFA_act_ and PLFA_fun_.

Regarding the different parameters measured in relation to the community of AMF, soil amendment with DOR-based biochar significantly increased NLFA_AMF_ in soil; nevertheless, soil contents of EE-GRSP and the percentage of AMF root colonization did not significantly vary (Table 1, Fig. 3). Soil contamination level was a significant factor for NLFA_AMF_, EE-GRSP and AMF root colonization. Soil NLFA-based abundance of AMF and AMF root colonization were significantly higher in soil “medium” compared with the other two soils; instead, the highest values for EE-GRSP were recorded in soil “high” throughout the experiment (Fig. 3). The highest soil contents for NLFA_AMF_ and EE-GRSP were obtained at time 60 days, while the percentage of AMF root colonization was the highest after 30 days of treatment; i.e., the factor soil treatment was significant for the three parameters. As expected, the inoculation of *F. mosseae* resulted effective at boosting NLFA_AMF_, soil content of EE-GRSP and AMF root colonization. However, the interaction between biochar and AMF inoculation was not significant for any of them (Table 1, Fig. 3). The interaction B × S × T × M showed to be significant (p < 0.001) only for EE-GRSP. Spearman correlation analyses showed that contents of NLFA_AMF_ and EE-GRSP were significantly positively correlated (ρ = 0.138, p = 0.009). Percentage of AMF root colonization did not significantly correlate with NLFA_AMF_ (ρ = 0.101, p = 0.058) nor with EE-GRSP contents (ρ = 0.027, p = 0.616).

### Evaluation of the factors pyrolysis temperature and application dose of the DOR-based biochar, soil contamination level, soil treatment time, *F. mosseae* inoculation and their interactions

In a second analysis, only the amended microcosms were considered in order to evaluate the effect of the factors biochar pyrolysis temperature (P) and biochar amendment dose (D) along with the soil contamination level (S), soil treatment time (T), *F. mosseae* inoculation (M) and their interactions on the different parameters considered in the study (Tables 2, 3, 4, Figs. 1, 2, 3). Both production temperature and application dose of DOR-based biochar significantly affected β-glucosidase and dehydrogenase activities, but opposite patterns were observed. While β-glucosidase activity was significantly higher after soil application of BC500 (compared with BC350) and at a dose of 2% (compared with a 5% dose), dehydrogenase was stimulated to a significantly higher extent by BC350 and by a dose of 5% (Table 2, Fig. 1). The factors biochar production temperature and application dose significantly interacted with soil contamination level for β-glucosidase (P × D × S, p < 0.001) and with soil contamination and soil treatment time for dehydrogenase (P × D × S × T, p < 0.001). The inoculation of *F. mosseae* did not significantly alter the β-glucosidase activity, but the interaction between AMF application and biochar pyrolysis temperature and application dose was significant (P × D × M, p < 0.05, Table 2, Fig. 1). Significantly lower levels of dehydrogenase activity were obtained in the *F. mosseae-*inoculated microcosms. The interaction between the factor AMF inoculation and dose of application, soil contamination and treatment time evaluated was significant for dehydrogenase (D × S × T × M, p < 0.001).

**Table 2:** Results of MANOVA and post-hoc analyses on the effect of the factors pyrolysis temperature of DOR-based biochar (350 and 500 °C), application dose of DOR-based biochar (2 and 5%), soil contamination level (“low”, “medium” and “high”), soil treatment time (30, 60 and 90 days), inoculation of *F. mosseae* (AMF) and their interactions on soil enzyme activities (β-glucosidase and dehydrogenase) considering only amended microcosms.

Factors	β-glucosidase^a^	Dehydrogenase^b^
**DOR pyrolysis temperature (P)**		
F^significance^ (df)^c^	10.81*** (1)	7.52*** (1)
Post-hoc test		
350 °C	1162.88 a	0.40 b
500 °C	1200.48 b	0.39 a
**Biochar application dose (D)**		
F^significance^ (df)^c^	51.80*** (1)	15.86*** (1)
Post-hoc test		
2%	1232.74 b	0.38 a
5%	1148.28 a	0.41 b
**Soil contamination level (S)**		
F^significance^ (df)^f^	46.46*** (2)	5044.91*** (2)
Post-hoc test		
Low	1436.14 b	0.45 b
Medium	1122.88 a	0.46 b
High	1229.23 a	0.14 a
**Soil treatment time (T)**		
F^significance^ (df)^f^	936.05*** (2)	22.84*** (2)
Post-hoc test		
30 days	1100.52 a	0.41 b
60 days	1191.51 b	0.42 b
90 days	1317.82 c	0.38 a
***F. mosseae* inoculation (M)**		
F^significance^ (df)^f^	0.51 (1)	18.17*** (1)
Post-hoc test		
No inoculation	1196.07 a	0.44 b
Inoculation	1183.59 a	0.38 a
Significant interactions	P × D*, P × M*, D × M*, S × T***, T × M***, P × D × S***, P × S × T*, P × D × M*, D × S × M***	D × S*, D × T***, D × M*, S × T***, S × M***, M × T*, P × D × T***, D × S × T***, D × S × M***, D × T × M***, S × T × M***, P × D × S × T***, D × S × T × M***

Median values for each variable at each factor level are also shown.

^a^For post-hoc tests, the values are expressed as µPNP (g soil dm)^−1^ (1 h)^−1^.

^b^For post-hoc tests, the values are expressed as μmol INTF (g soil dm)^−1^ (1 h)^−1^.

^c^F-value^significance^ (degrees of freedom).

For MANOVA analyses, F-values in bold denote statistical significance (p ≤ 0.05), significance levels are shown at *p < 0.05, **p < 0.01 and ***p < 0.001; for post-hoc Tukey’s HSD tests, median values followed by different letters are significantly different (p < 0.05).

**Table 3:** Results of MANOVA and post-hoc analyses on the effect of the factors pyrolysis temperature of the DOR-based biochar (350 and 500 °C), application dose of DOR-based biochar (2 and 5%), soil contamination level (“low”, “medium” and “high”), soil treatment time (30, 60 and 90 days) and inoculation of *F. mosseae* (AMF) and their interactions on PLFA-based abundance of the different microbial groups considering only amended microcosms.

Factors	PLFA_tot_^a^	PLFA_bac_^a^	PLFA_Gram+_^a^	PLFA_Gram-_^a^	PLFA_act_^a^	PLFA_fun_^a^
**DOR pyrolysis temperature (P)**						
F^significance^ (df)^b^	0.32 (1)	0.10 (1)	2.64 (1)	0.87 (1)	1.29 (1)	16.03*** (1)
Post-hoc test						
350 °C	13.55 a	9.22 a	3.32 a	4.10 a	1.23 a	0.29 b
500 °C	14.01 a	9.69 a	3.21 a	4.08 a	1.25 a	0.25 a
**Biochar application dose (D)**						
F^significance^ (df)^b^	38.62*** (1)	38.93*** (1)	11.87*** (1)	47.26*** (1)	0.41 (1)	65.38*** (1)
Post-hoc						
2%	13.57 a	9.28 a	3.09 a	4.01 a	1.23 a	0.23 a
5%	14.01 b	9.95 b	3.37 b	4.23 b	1.27 a	0.30 b
**Soil contamination level (S)**						
F^significance^ (df)^b^	632.59*** (2)	908.16*** (2)	502.64*** (2)	524.27*** (2)	931.66*** (2)	181.50*** (2)
Post-hoc test						
Low	15.44 b	10.78 b	4.21 c	4.42 b	1.56 c	0.20 b
Medium	15.41 b	11.16 c	3.92 b	4.99 c	1.32 b	0.38 c
High	8.00 a	5.04 a	1.73 a	2.41 a	0.58 a	0.16 a
**Soil treatment time (T)**						
F^significance^ (df)^b^	35.34*** (2)	69.16*** (2)	0.20 (2)	101.52*** (2)	6.87*** (2)	88.32*** (2)
Post-hoc test						
30 days	15.22 c	10.44 c	3.31 a	5.34 c	1.33 b	0.35 b
60 days	13.55 b	9.32 b	3.01 a	3.93 b	1.26 ab	0.31 b
90 days	12.65 a	8.95 a	4.02 a	3.71 a	1.23 a	0.20 a
***F. mosseae* inoculation (M)**						
F^significance^ (df)^b^	13.15*** (1)	5.86*** (1)	0.29 (1)	27.47*** (1)	2.24 (1)	24.01*** (1)
Post-hoc test						
No inoculation	13.03 a	9.93 b	3.18 a	3.90 a	1.22 a	0.25 a
Inoculation	14.13 b	9.09 a	3.33 a	4.41 b	1.31 a	0.30 b
Significant interactions	P × M***, D × S***, D × T*, S × T***, T × M*, P × D × S*, P × S × M***, D × S × T***, D × S × M*, S × T × M*, P × D × S × T* P × D × T × M*,	P × T***, P × M***,D × S***, S × T***, S × M***, T × M***, P × D × M*, P × D × S*, D × S × T***, D × S × M***, S × T × M***, P × D × S × T*, P × D × T × M*	P × D ***, P × T*, P × M*, D × S***, D × M***, S × T***, S × M***, T × M*, P × D × S*, D × S × T***, D × S × M***, S × T × M***, P × D × S × M*, P × D × T × M*	P × T*, P × M***, D × S *, D × T*, S × T***, P × S × T*, D × S × T*, S × T × M *, P × D × T × M*	P × M***, D × S*, S × T***, S × M*, D × S × T*, P × S × M*	P × D*, P × M***, D × S***, S × T***, S × M*, D × S × M*, P × D × T × M***

Median values for each variable at each factor level are also shown.

^a^For post-hoc tests, the values are expressed as µg PLFA (g soil dm)^-1^.

^b^F-value^significance^ (degrees of freedom).

For MANOVA analyses, F-values in bold denote statistical significance (p ≤ 0.05), significance levels are shown at *p < 0.05, **p < 0.01 and ***p < 0.001; for post-hoc Tukey’s HSD tests, median values followed by different letters are significantly different (p < 0.05).

**Table 4:** Results of MANOVA and post-hoc analyses on the effect of the factors pyrolysis temperature of the DOR-based biochar (350 and 500 °C), application dose of the DOR-based biochar (2 and 5%), soil contamination level (“low”, “medium” and “high”), soil treatment time (30, 60 and 90 days) and inoculation of *F. mosseae* (AMF) and their interactions on NLFA-based abundance of AMF, easily extractable glomalin-related soil content (EE-GRSP) and AMF-root colonization rate considering only amended microcosms.

Factors	NLFA_AMF_^a^	EE-GRSP^b^	AMF-root colonization^c^
**DOR pyrolysis temperature (P)**			
F^significance^ (df)^d^	1.33 (1)	3.47 (1)	2.41 (1)
Post-hoc test			
350 °C	0.83 a	0.160 a	6.00 a
500 °C	0.72 a	0.163 a	6.00 a
**Biochar application dose (D)**			
F^significance^ (df)^d^	4.53* (1)	8.21*** (1)	42.41*** (1)
Post-hoc test			
2%	0.72 a	0.160 a	5.50 a
5%	0.78 b	0.174 b	6.00 b
**Soil contamination level (S)**			
F^significance^ (df)^d^	1268.44*** (2)	3810.96*** (2)	79.86*** (2)
Post-hoc test			
Low	0.88 b	0.151 b	7.00 c
Medium	1.40 c	0.112 a	5.55 b
High	0.17 a	0.176 c	5.00 a
**Soil treatment time (T)**			
F^significance^ (df)^d^	100.44*** (2)	1264.24*** (2)	971.85*** (2)
Post-hoc test			
30 days	0.65 a	0.146 a	13.00 c
60 days	1.26 c	0.181 c	6.00 b
90 days	0.70 b	0.155 b	3.55 a
***F. mosseae* inoculation (M)**			
F^significance^ (df)^d^	26.54*** (1)	4259.38*** (1)	48.97*** (1)
Post-hoc test			
No inoculation	0.73 a	0.159 a	5.00 a
Inoculation	0.81 b	0.164 b	7.00 b
Significant interactions	P × S***, S × T***, S × M***, T × M*, S × T × M***	S × M***, S × T***, T × M***, P × S × M*, P × T × M*, S × T × M***, P × S × T × M***	P × D*, P × T*, D × S***, D × T***, D × M***, S × T***, S × M***, T × M***, P × S × T*, P × D × T***, P × T × M***, D × S × T***, D × T × M***, S × T × M*, P × D × S × T***, P × D × S × M*, P × S × T × M***, P × D × T × M***, D × S × T × M***

Median values for each variable at each factor level are also shown.

^a^For post-hoc tests, the values are expressed as µg NLFA (g soil dm)^−1^.

^b^For post-hoc tests, the values are expressed as g (g soil dm)^−1^.

^c^For post-hoc tests, the values are expressed as %.

^d^F-value^significance^ (degrees of freedom).

For MANOVA analyses, F-values in bold denote statistical significance (p ≤ 0.05), significance levels are shown at *p < 0.05, **p < 0.01 and ***p < 0.001; for post-hoc Tukey’s HSD tests, median values followed by different letters are significantly different (p < 0.05).

The factor DOR pyrolysis temperature did not have a significant effect on the abundance of the different PLFA-based microbial fractions, except for fungi (PLFA_fun_ increased after soil BC350 application in comparison with BC500 addition, Table 3, Fig. 2). Instead, the factor dose was significant for all PLFA-based microbial groups, except Actinobacteria, with the highest microbial abundances recorded at a dose of 5% (Table 3, Fig. 2). The factor dose showed to significantly affect the soil microbial abundance not only by itself, but also by its interaction with the factors soil contamination level and soil treatment time for all the microbial groups except fungi (D × S × T, p < 0.05, Table 3, Fig. 2). Soil inoculation with *F. mosseae* significantly increased total biomass, Gram- and fungal PLFA contents, whilst bacterial contents decreased (Table 3, Fig. 2). For PLFA_tot_, PLFA_bac_, PLFA_Gram+_, PLFA_Gram-_ and PLFA_fun_, the interactions P × D × S × M (p < 0.05) or P × D × T × M (p < 0.001) showed to be significant (Table 3).

DOR pyrolysis temperature did not significantly influence soil contents of mycorrhizal NLFA marker and glomalin nor the percentages of AMF root colonization (Table 4, Fig. 3). However, increased values for all these parameters were observed following DOR-based biochar application at a dose of 5% in comparison with the 2% dose. The interaction of the factors P × D × T × M (p < 0.001) was significant only for AMF root colonization rate. The presence of *F. mosseae* in the rhizosphere of wheat plants significantly boosted NLFA markers for AMF, soil glomalin contents and the AMF root colonization percentage (Table 4, Fig. 3). When only amended microcosms are considered, soil EE-GRSP contents and NLFA_AMF_ were significantly positively correlated (Spearman rank correlation; ρ = 0.158, p = 0.008). However, mycorrhization rates were not significantly correlated with NLFA_AMF_ (ρ = 0.113, p = 0.058) nor with soil EE-GRSP contents (ρ = 0.0155, p = 0.795).

## Discussion

Studies assessing the effectiveness of treatments based on the soil application of organic amendments to recover metal-contaminated soils should consider not only the effect of such treatments in the reduction of metal bioavailability and/or mobility, but also their concomitant potential to improve the status of microbial communities since microbes are indispensable in maintaining healthy and fertile soils. In this way, we proposed in the present work to investigate the potential of DOR-based biochar application along with AMF inoculation (using the fungus *F. mosseae*) in recovering the microbial communities of three soils presenting a metal-pollution gradient as a complementary study to the previous insights about the DOR-based biochar impact on soil metal behavior and plant nutrition status reported recently by Vejvodová et al*.*^15^.

Our work was mainly focused on monitoring soil potential enzyme activities and community abundance since these parameters have been regarded as useful indicators in assessing the impact of biochar on soil quality^29^. In fact, soil enzymatic activities have shown to respond faster than other soil variables to soil management and disturbance processes^30^. In our experiment, the significantly highest values for β-glucosidase and dehydrogenase were recorded in the least contaminated soil, which is in line with other works showing that increased metal pollution negatively impacts microbial functionality^31^. Our preliminary expectations, based on studies such as that of Pokharel et al*.*^32^, were to find an increase in microbial activities after soil amendment with biochar, especially in the most contaminated soil. However, in our study, DOR-based biochar did not boost β-glucosidase activity nor dehydrogenase; in fact, β-glucosidase was inhibited. Although soil amendment with biochar tends to have a positive effect on enzyme activities, decreased values in β-glucosidase activity followed soil application of biochar derived from sewage sludge at rates of 4% and 8% (w/w) were reported^33^. In our experiment, the significance of the interaction between biochar application and soil contamination level points out that the effect of biochar on this enzyme is dependent on the pollution level of the soil. On the other hand, temperature of pyrolysis is a key parameter in controlling the properties of biochar, especially to designate the short or long-term availabilities of C in soil as well as the accumulation of mineral nutrients and/or OM combustion residue^34^. As demonstrated by Vejvodová et al.^15^, BC500 was richer than BC350 in C and some other mineral nutrients (except N). In our study, pyrolytic temperature of biochar significantly influenced both enzyme activities, although a clear pattern was not observed. The highest values for β-glucosidase were found after the application of BC500, while dehydrogenase was recorded at the highest extent in the microcosms amended with BC350. We also found that β-glucosidase was inhibited to a higher extent after applying DOR-based biochar at a dose of 5% compared with the 2% dose. Conversely, dehydrogenase activity was significantly higher after soil amendment at a dose of 5% in comparison with the 2%. These findings are in line with other works stating that soil enzyme activities respond to biochar application in various ways, depending on the type of enzyme and biochar, the biochar application rate and soil properties^32^. In this way, biochars might affect soil microbial activities by means of the following mechanisms, among others: (i) enriching soil with specific nutrients and substrates, which induces the microbial production of enzymes; (ii) adsorbing extracellular enzymes and/or substrates on the surface or blocking the reaction site of enzymes in a way that regulates their affinity for substrates; (iii) indirectly, by producing shifts in soil physicochemical properties; and (iv) releasing some small molecules that may act as allosteric regulators or inhibitors of specific enzymes^35^.

Interestingly, although soil amendment with DOR-based biochar decreased β-glucosidase activity, the inoculation of *F. mosseae* had a significantly positive effect on this activity. Likewise, these two factors significantly interacted to influence β-glucosidase, which points out the suitability of incorporating AMF in recovery strategies of metal contaminated soils that involve the application of biochar^21^. Additionally, *F. mosseae* inoculation led to a significant increase in AMF hyphal density (as demonstrated by increased EE-GRSP contents^36^), which would have benefited the stability of soil aggregates and had a beneficial effect on β-glucosidase. Thereby, combining DOR-based biochar and AMF inoculation may result in a synergistic effect based on the high C-substances input supplied by adding DOR-based biochar along with the better stability of soil aggregates provided by AMF^24,28,37^. This result might also suggest that *F. mosseae* stimulates indirectly the microbial activity by changing the patterns of root exudates, as previously reported^38^.

FA have been broadly used to create a profile of fingerprints of the community structure using biomarkers for specific groups of microorganisms^39,40^ and are useful indicators of soil attributes to evaluate the recovery of the ecological soil functions, as Covino et al.^41^ suggested. In our study, the abundance of the different microbial fractions (PLFA_tot_, PLFA_bac_, PLFA_Gram+_, PLFA_Gram-_, PLFA_act_ and PLFA_fun_) was significantly positively affected by DOR-based biochar. Furthermore, the 5% dose boosted microbial abundances to a significantly higher extent than the 2% dose did. The better response of microbial populations to a higher dose of application of DOR-based biochar could indicate an improvement of the physical parameters of soils (i.e., porosity, humidity and aeration) and nutrient availability, which leads to a favorable habitat for microbial communities, as indicated by El-Naggar et al.^42^. It has also been proposed that the highly porous structure of biochar could provide a congenial habitat niche for the growth of soil microorganisms^43^. Likewise, the decrease found in Cd, Pb and Zn mobility after the application of DOR-based biochar to soils “low” and “medium”^15^, clearly shows the beneficial impact of this amendment on microbial communities by increasing pH and its feedback on nutrient availability and potential reduction of metal toxicity^44^. Throughout our experiment, the highest PLFA values for the different microbial groups were obtained in the “low” and “medium” soils, which, according to García-Sánchez et al.^45^, is indicative of the better efficiency of DOR-based biochar in metal-stabilization in moderately contaminated soils in comparison with the extremely contaminated ones. Additionally, it is worth noting that abundances of the different microbial groups were never the highest in the most contaminated soil, even after biochar amendment, which shows the detrimental effect that metals exert on microbial abundances^31^. Although BC500 was richer in some nutrients, microbial communities were not affected by these differences in terms of abundance. On the other hand, it is reasonable to argue that nutrients supplied by biochar decreased in soil microcosms over time, which would explain the decreasing microbial abundances obtained with longer experiment times. The inoculation of *F. mosseae* had an ambiguous impact on microbial abundances since Gram− bacteria and fungal populations were positively affected, in contrast with the decline found in Gram+ bacteria and Actinobacteria groups. This suppressive effect has been already reported by García-Sánchez et al.^28^. The reason for this effect may be the production of antagonistic metabolites by AM fungal exudates, as it has been reported for *Glomus* sp.^46^.

Previous studies have argued that biochar benefits the growth of AMF by increasing the availability of micronutrients, changing soil physical and chemical properties, promoting spore germination and hyphal branching^47^. In our study, although NLFA_AMF_ contents increased after the application of DOR-based biochar, neither levels of EE-GRSP in soil nor percentages of AMF root colonization were higher after soil application of DOR-based biochar. A possible explanation for this contradictory result may be that the susceptibility of plant roots to be colonized depends on the plant species, as some authors have suggested^20,28^. This argument could also explain the lack of a positive correlation between AMF root colonization and soil contents of NLFA_AMF_ or EE-GRSP in our experiment. The reduction in EE-GRSP contents could be also due to the presence of certain chelating humic substances supplied by DOR-based biochar^36^. On the other hand, when only amended microcosms were considered, the 5% dose significantly favored the NLFA_AMF_, EE-GRSP content and AMF root colonization in comparison with the 2% dose. This finding points out that the key factor influencing the effect of DOR-based biochar on soil AMF community seems to be its dose application. Despite the use of biochar, the lowest AMF root colonization rates were obtained in the most contaminated soil, presenting tenfold the contents of some metals of the soil “medium”, which highlights the negative effect that metals exert on AMF development^23^. As expected, soil inoculation with *F. mosseae* led to significantly higher soil contents of NLFA_AMF_ and EE-GRSP and higher AMF root colonization values. It is worth noting that we found a significantly positive correlation between soil contents of the NLFA 16:1ω5 and EE-GRSP throughout our experiment. But interestingly, these two biomarkers have been related to different compartments of AMF community in soil; NLFA 16:1ω5 represents storage lipids in AMF spores, while EE-GRSP is rather a marker of the mycorrhizal mycelium in soil^48^. Therefore, although the quantification of the NLFA 16:1ω5 in soil may not represent the actual active mycorrhizal population, its assessment has been considered as complementary to that of EE-GRSP to achieve an integrative analysis of soil AMF community^48^. In any case, the NLFA 16:1ω5 has been shown to be a more reliable marker of soil AMF population than the PLFA 16:1ω5^49^. In our study, the highest values in soil NLFA_AMF_ and EE-GRSP contents were found after 60 days, while rates of root colonization were the highest at time 30 days. After that sampling time, colonization rates decreased, probably as the result of a reduced physical growing space for AMF.

## Conclusions

The feasibility of the soil application of DOR-based biochar alone or in combination with *F. mosseae* to enhance the status of microbial communities (in terms of potential functionality and abundance) inhabiting three soils with a gradient of metal contamination was evaluated to complement our previous study^15^. We showed that microbial enzyme activities (β-glucosidase and dehydrogenase) were differently affected by the specific properties of DOR-based biochar, which resulted to be dependent on the temperature of production (350 vs. 500 °C). The application dose of DOR-based biochar induced significant changes in the soil microbial status, especially at boosting the FA-based abundance of all microbial groups. The efficiency of DOR-based biochar in improving soil microbial status was higher when it was applied to the moderately contaminated soil. Soil inoculation with *F. mosseae* led to a proliferation of AMF community, increased mycorrhization rates of wheat plants and higher levels of β-glucosidase activity. Interestingly, although the *F. mosseae* inoculation itself had a limited effect on boosting PLFA-based microbial abundances, it significantly interacted with the soil amendment with DOR-based biochar to favor the microbial proliferation. Therefore, our results postulates that the soil application of DOR-based biochar in combination with AMF inoculation is a suitable approach to improve the microbial status of moderately metal contaminated soils at the short term. We recognize that these conclusions are limited to the soils assayed and the experimental conditions used. In this way, further studies evaluating this approach at the long term are needed under different metal-contaminated environments.

## Materials and methods

### Site description and tested soils

As explained by Vejvodová et al*.*^15^, the chronically contaminated soils used in this experiment were collected in the vicinity of Trhové Dušníky village, which is located in the Příbam district of the Czech Republic. A detailed description of the location, including a detailed map of the area, is available elsewhere^50^. The intense regional mining and smelting activities in this area during the last centuries have resulted in soil contamination at different rates with metals such as As, Cd, Pb and Zn as the result of polymetallic mineral depositions^51^. In our experiment, three sites differing in the levels of metal concentration were selected^15^ and identified as “low” (slightly polluted), “medium” (moderately polluted) and “high” (highly polluted). The locations chosen were the following: soil “low” (49° 43′ 15.730″ N; 13° 58′ 33.126″ E), soil “medium” (49° 42′ 43.450″ N; 13° 59′ 7.615″ E), and soil “high” (49° 43′ 9.353″ N; 14° 0′49.828″ E). The three soils are at the same location under the same environmental and climatic conditions and are thus comparable. Soil samples from each site were collected at a depth of 0–20 cm, immediately homogenized, air-dried at room temperature and finally passed through a 5 mm mesh sieve. Soils were stored in polythene bags and kept at room temperature until their use. “Low” and “medium” soils were classified as Cambisol and the “high” soil as Fluvisol and they differed on their their physicochemical characteristics which have previously been reported^15^.

### Biochar preparation

The biochar used in this study was produced from dry olive residue (DOR), which was supplied by the olive oil manufacturer Sierra Sur S.A. (Granada, Spain). The DOR was sieved, sterilized by autoclaving it three times and stored at 4 °C prior to use. Its basic characteristics have previously been described by Siles et al*.*^10^. The biochar was produced in laboratory conditions using a pyrolytic furnace Carbolite 301 (Carbolite Gero, Great Britain). The pyrolysis was performed in an electrically heated quartz tube for 25 min at the target temperatures of 350 °C (from now on referred to as BC350) and 500 °C (BC500) in the presence of nitrogen (N_2_ flow of 4.5 L per min). The DOR-based biochar was immediately homogenized after pyrolysis and physicochemical composition was determined and published elsewhere^15^.

### Inoculum of arbuscular mycorrhizal fungus

The AM fungus used in this experiment was *Funneliformis mosseae* (formerly named as *Glomus mosseae*) due to its resistance and adaptation to metal-contaminated soils as our previous studies showed^15,28^. The mycorrhizal inoculum was a mixture of rhizosphere soil containing spores, hyphae and mycorrhizal root fragments. This material was obtained by using trap pot cultures of *Medicago sativa* L., consisting of soil, spores, mycelia and colonized root fragments (10 sporocarps g^−1^, with 1–5 spores per sporocarp).

### Experimental design and set up

The experimental design consisted of a factorial system including the following factors: (i) soil application rate of the DOR-based biochar, comprising the levels 0% (control), 2% and 5%; (ii) pyrolysis temperature of the DOR-based biochar, which includes the temperatures 350 °C and 500 °C; (iii) soil metal contamination level, comprising the levels “low” (slightly contaminated), “medium” (moderately contaminated) and “high” (highly contaminated); (iv) soil treatment time, including the levels 30, 60 and 90 days; and (v) AMF inoculation, comprising the levels application or not of *F. mosseae*. The experiment was set up in identical 0.3 L polypropylene pots. Approximately, 300 g of “low”, “medium” or “high” multi-contaminated soil was placed into each pot. BC350 and BC500 were applied and manually mixed with the soil to reach the concentrations of 2% and 5% (w/w). One half of the amended pots were inoculated with *F. mosseae* by adding 8 g of inoculum, as suggested by Vejvodová et al.^15^; meanwhile, the other half received the same weight of inoculum filtrate (Whatman no. 1 filter paper) containing soil microbiota free of AM fungal propagules. An initial irrigation was realized gravitationally in order to reach the given value of the soil water holding capacity (WHC = 60%). Soil samples with or without application of DOR-based biochar and *F. mosseae* were also set up and used as controls. Five replicates were established for each treatment. In total, the experiment consisted of 325 pot microcosms (5 factors × 13 factor levels × 5 replicates).

The use of plants in the present study complies with international/institutional guidelines. Before planting, wheat seeds (*Triticum aestivum* L.) were surface disinfected by immersion in 2% (v/v) hydrogen peroxide for 8 min^52^. Seed germination was carried out at 25 ºC in trays containing vermiculite as substrate for two weeks. Afterward, one 15-day old wheat plant was planted in each pot. The experiment was run in greenhouse conditions (supplementary light 25/19 °C and 50% relative humidity), as previously described^15^, and microcosms were regularly watered in order to maintain the initial moisture conditions. Soil microcosms were destructively sampled after 30, 60 and 90 days. Soil from each pot at each time sampling was collected, homogenized, sieved (2 mm mesh) and subdivided into two subsamples. One subsample was kept at 4 °C for biochemical analyses and the other one was frozen at − 80 °C and then freeze-dried for FA analyses.

### Soil enzyme activities

Dehydrogenase activity (EC 1.1) was measured using 0.5% INT (2-p-iodofenil-3-p-nitrofenil-5-feniltetrazolio) as substrate following the methodology of Camiñas et al.^53^. 1 g of soil was incubated with 2 mL of the substrate 2-p-iodophenyl-3-p-nitrophenyl-5-phenyltetrazolium (INT) 0.5% and 1.5 mL of 1 M Tris–HCl buffer pH 7.5 for 1 h at 40 °C. Next, the iodonitrotetrazolium formazan (INTF) formed was extracted with a 1:1 (v:v) mixture of ethanol and dimethyl-formamide, and its absorbance at 490 nm was measured. β-glucosidase activity (EC 3.2.1.21) was determined using 0.05 M PNG (p-nitrophenyl-β-d-glucopyranoside) as substrate following the procedure described by Eivazi et al.^54^. 1 g of dry soil was mixed with 5 mL of a solution of p-nitrophenyl β-d-glucopyranoside dissolved at a concentration of 0.025 M in buffer MUB (0.1 M, pH 6), and incubated at 37 °C for 2 h. The substrate used was transformed into p-nitrophenol due to the action of β-glucosidase, and the concentration of this compound was determined at 400 nm after the addition of 1 mL of 0.5 M CaCl2 and 4 mL of 0.1 M THAM buffer pH 12.

### Fatty acid analysis

Soil samples were firstly extracted using a mixture of chloroform–methanol-phosphate buffer (1:2:0.8; v/v/v), according to Bligh and Dyer^55^. Thereafter, the lipids were fractioned into neutral lipids (NLFAs), glycolipids and polar lipids (PLFAs), using solid-phase extraction cartridges (LiChrolut Si-60, Merck, White-house Station, USA), by elution with chloroform, acetone and methanol, respectively. PLFAs and NLFAs were then subjected to mild alkaline methanolysis, as described by Šnajdr et al*.*^56^, and free methyl esters of NLFAs and PLFAs were analyzed by gas chromatography-mass spectrometry (450-GC, 240-MS ion trap detector, Varian, Walnut Creek, CA), following the procedure described by Ref.^57^.

Bacterial biomass (PLFA_bact_) was quantified as a sum of i14:0, i15:0, a15:0, 16:1ω5, 16:1ω7, 16:1ω9, 10Me-16:0, i16:0, i17:0, a17:0, cy17:0, 17:0, 10Me-17:0, 18:1ω7, 10Me-18:0 and cy19:00. Actinobacteria abundance (PLFA_act_) was determined according to 10Me-16:0, 10Me-17:0 and 10Me-18:0. Fungal biomass (PLFA_fun_) was quantified based on the content of 18:2ω6,9^56^. The ∑ PLFAs was used to estimate the total microbial biomass (PLFA_tot_). The NLFA 16:1ω5 was assigned as a marker for the quantification of AM fungal population^40^.

### Root mycorrhization rate and soil glomalin content

The percentage of mycorrhizal fungi root length infected was performed using a microscope (20–40×) after clearing a portion of the roots with 3% H_2_O_2_ (v/v) and 10% KOH (w/v) and staining in a 0.05% solution of fuchsine blue in lactic acid (w/v). The gridline intercept method^58^ was used for determining the percentage of AM root colonization.

To determine contents of easily extractable glomalin-related soil protein (EE-GRSP) in soil, 1 g of soil was firstly suspended in 8 mL of citrate buffer (20 mM, pH 7.0) and the suspension was then autoclaved at 121 °C for 30 min Wright and Upadhyaya^59^, centrifuged at 10,000×*g* for 15 min and filtered through Whatman no. 1 filter paper. EE-GRSP contents in the filtrates were determined by the Bradford protein assay (Bio-Rad Protein Assay; Bio-Rad Labs) with bovine serum albumin as standard^59^.

### Statistical analyses

The significance of the factors evaluated here and their interactions on soil enzyme activities, PLFA- and NLFA-based microbial abundance, EE-GRSP, and percentage of AMF root colonization were analyzed by using two separate multifactor ANOVAs (analysis of variance). A first four-way ANOVA was conducted considering the factors DOR-based biochar application (levels of the factor: application or not), soil contamination level (low, medium and high), soil treatment time (30, 60 and 90 days), AMF inoculation (yes or not) and their interactions. A second five-way ANOVA considered the factors DOR pyrolysis temperature (300 and 500 °C), application dose of DOR-based biochar (2 and 5%), soil contamination level, soil treatment time, AMF inoculation and their interactions. Whenever ANOVA resulted in significant results, Tukey’s HSD (honest significance difference) post-hoc test was used for multiple comparisons of means at a 95% confidence interval. Normality and heteroscedasticity of data were tested using the Shapiro–Wilk’s and Levene tests, respectively. In case that one of those conditions was not met, the values were transformed by multiplying them by a constant and applying afterwards natural logarithms. Spearman rank correlation analyses were used to find significant links between some of the parameters evaluated in the study. Data visualizations were performed using the R package ggplot2 and CorelDRAW ver. 2020^60^.
